# Phenological divergence between flowering plants and flower-visiting Lepidoptera under climate change across Europe

**DOI:** 10.1098/rspb.2026.1381

**Published:** 2026-11-04

**Authors:** Yanru Huang, Chaoyang Wu, Wenjiang Huang, Yang Liu, Man Qi, Jie Bai, Yingying Dong, Samuel J. L. Gascoigne, Philippe Ciais, Josep Peñuelas, Rob Salguero-Gomez

**Affiliations:** ^1^ State Key Laboratory of Remote Sensing and Digital Earth, Aerospace Information Research Institute, Chinese Academy of Sciences, Beijing 100094, People's Republic of China; ^2^ University of Chinese Academy of Sciences, Beijing 100049, People's Republic of China; ^3^ Department of Biology, University of Oxford, Oxford OX1 3SZ, UK; ^4^ The Key Laboratory of Land Surface Pattern and Simulation, Institute of Geographical Sciences and Natural Resources Research, Chinese Academy of Sciences, Beijing 100101, People's Republic of China; ^5^ State Key Laboratory of Information Engineering in Surveying, Mapping and Remote Sensing, Wuhan University, Wuhan 430079, People's Republic of China; ^6^ University of Electronic Science and Technology of China, Chengdu 611731, People's Republic of China; ^7^ Laboratoire des Sciences du Climat et de l'Environnement, IPSL-LSCE CEA CNRS UVSQ, 91191, Gif-sur-Yvette, France; ^8^ CSIC, Global Ecology Unit CREAF-CSIC-UAB, Bellaterra, Barcelona 08193, Catalonia, Spain; ^9^ CREAF, Cerdanyola del Valles, Barcelona 08193, Catalonia, Spain

**Keywords:** climate change, phenology, environmental sensitivity, phenological mismatch, plant–insect interactions

## Abstract

Climate change is altering life-cycle timing and may disrupt coordinated interactions between flowering plants and flower-visiting insects. Using Global Biodiversity Information Facility observation records from Europe between 2000 and 2024, we compiled a continental-scale phenological dataset covering flowering periods of 707 angiosperm species and adult activity periods of 620 Lepidoptera species. We extracted the 10th, 50th and 90th percentiles and activity-window width and used linear mixed-effects models to analyse spatio-temporal trends. The results showed that plant phenology advanced significantly, with the 10th and 50th percentiles shifting earlier by −0.597 and −0.507 d yr⁻¹, respectively, whereas the 90th percentile showed no significant change; flowering-window width increased by 0.361 d yr⁻¹. By contrast, Lepidoptera phenology advanced evenly across the season, with the 10th, 50th and 90th percentiles shifting by −0.137, −0.153 and −0.155 d yr⁻¹, respectively, while activity-window width showed no significant change. Because flowering plants advanced faster than Lepidoptera in the early and middle season, plant–insect phenological overlap declined at 0.340 d yr⁻¹, indicating increasing mismatch across Europe. Further analyses identified temperature as the dominant driver of phenological advance in both groups, but flowering plants and Lepidoptera differed in their responses to other climatic factors. These results suggest that climate change is weakening phenological synchrony between flowering plants and flower-visiting Lepidoptera, with potential consequences for pollination and ecosystem functioning.

## 1. Introduction

The timing of recurrent biological events during the life cycle of an organism (e.g. maturation, reproduction and dispersal) is fundamental to its persistence [1]. Species phenologies are key to the maintenance of entire communities [2] and their ecosystem services [3]. Important phenological shifts, however, have been reported worldwide in recent decades [4]. For example, global warming has advanced the date of laying of great tits (*Parus major*) by more than two weeks in the past six decades [5], and the start of the growing season on the Qinghai–Tibetan Plateau has advanced by nearly 10 days in the past two decades [6].

Phenological adjustments under climate change represent organisms' responses to external environmental drivers [7,8] in concert with their intrinsic physiological–biochemical state [9–12]. Not all phenological shifts, however, are equal, nor do they have the same consequences. The direction and intensity of phenological shifts vary considerably across taxa [13,14], locations [15] and environments [16]. Importantly, species within a community can have different phenological shifts in response to the same climatic driver, thereby disrupting food webs [17]. Consequently, climate change may impede the temporal and spatial synchrony of biological interactions, potentially contributing to imbalances in trophic relationships [18].

The phenological synchrony between plants and insects at a given location ensures the match between the supply and demand of resources. Insect activity often coincides with specific stages of plant development in order to track resource availability and quality [19]. Phenological asynchrony emerges when the timing of key life-cycle events shifts at different rates and/or in different directions [20] (e.g. sooner or later in the growing season than historically). This phenomenon may have substantial ecological consequences, including reduced reproductive fitness [21,22] and increased mortality [23] in affected species, and may further increase the risk of local population declines or even local extinctions through cascading effects [24]. Indeed, under future warming scenarios (approx. 1.5–6.2°C), it has been estimated that 17–50% of pollinating insect species could become temporally mismatched with the flowering plant species they have historically visited, potentially reducing overlap with previously used floral resources [25]. Eighty-four percent of crops cultivated in Europe directly depend on insect-mediated pollination for their yield [26]. Thus, a potential mismatch between the phenologies of flowering plants and flower-visiting insects poses an important threat to the stability of both ecosystems and livelihoods.

Previous studies on plant–insect phenological synchrony have used a range of approaches [27], including manipulative experiments [28], field observations [29], broad-scale syntheses [30–32] and, increasingly, large biodiversity databases [33,34]. These studies have substantially advanced our understanding of phenological mismatch under climate change, but integrated comparisons of plant and insect phenology across broad spatial extents and long time periods remain limited, particularly when both are quantified within a common observational framework [35]. This challenge is especially pronounced for insects, whose diverse life histories and uneven monitoring coverage complicate large-scale assessments of seasonal activity. In this context, occurrence records archived by the Global Biodiversity Information Facility (GBIF) provide a valuable resource for reconstructing multi-species phenology across regions and years [36,37]. Integrating GBIF-based multi-species records can enable a more comprehensive assessment of synchrony between plant flowering and flower-visiting insect activity across Europe.

In this study, we used GBIF observation records (figure 1) to investigate the spatio-temporal variation in angiosperm flowering phenology and adult Lepidoptera activity phenology across Europe during 2000–2024. The data-processing workflow is shown in the supplementary material, figure S1. We used three quantile-based metrics, the 10th, 50th and 90th percentiles, to characterize early-, middle- and late-season phenological stages and structured the analysis around three main components. First, at the continental scale, we quantified long-term trends in plant flowering phenology and Lepidoptera activity phenology. Second, at the grid scale (1° × 1° latitude–longitude), we compared the spatial patterns of phenological change in flowering plants and Lepidoptera and evaluated differences in their temporal overlap. Third, using gridded environmental data from E-OBS, we analysed differences in the phenological sensitivities of flowering plants and Lepidoptera to changes in environmental factors and identified potential drivers of changes in their temporal matching.

**Figure 1.**
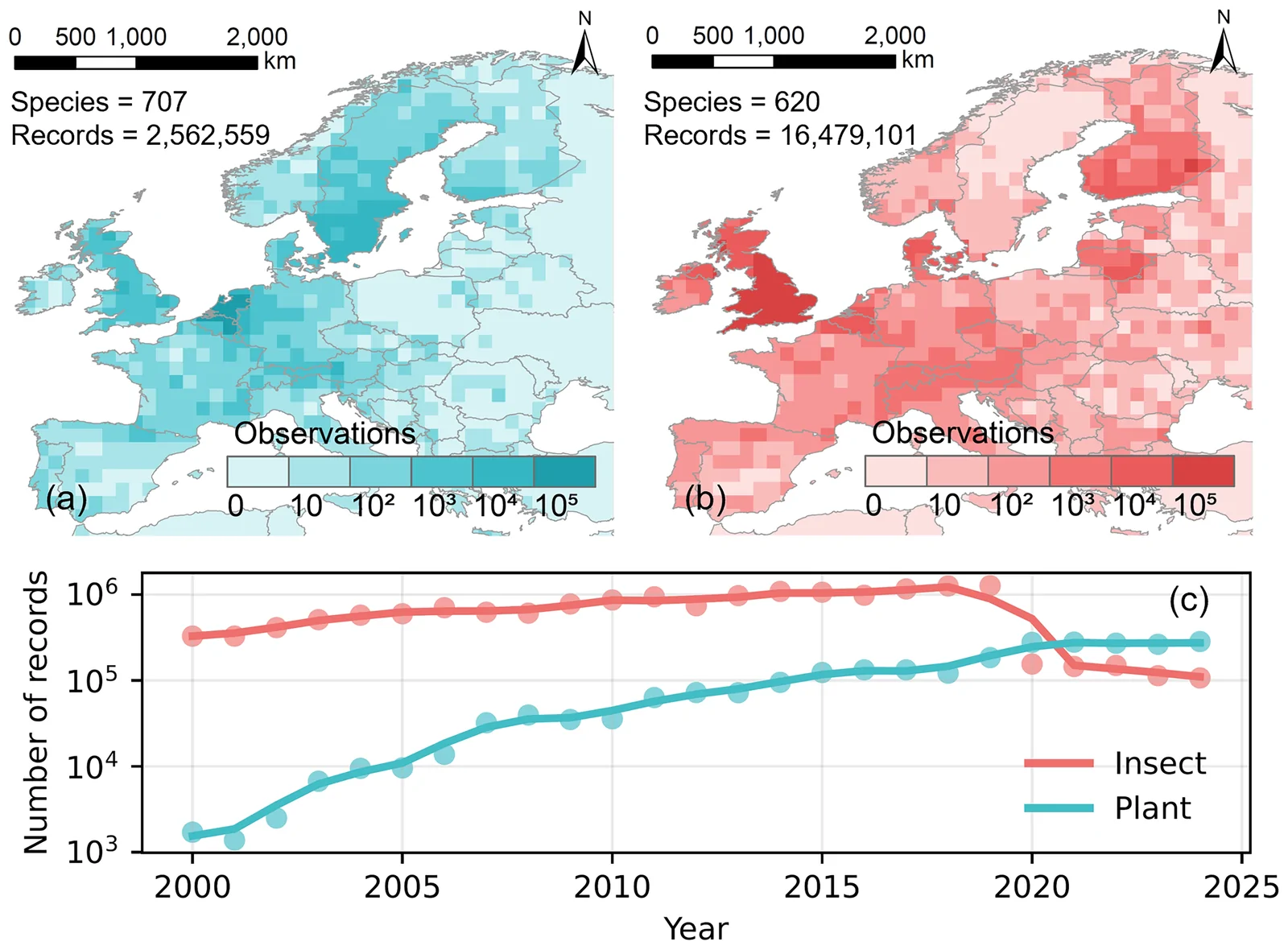
Spatial and temporal distributions of filtered flowering-plant and Lepidoptera observation records from GBIF across Europe during 2000–2024. (a) Flowering-stage records of angiosperms, (b) adult-stage records of Lepidoptera and (c) annual counts of flowering-plant and Lepidoptera observation records.

## 2. Methods

### (a) European flowering-plant and Lepidoptera occurrence records and filtering

To characterize spatio-temporal variation in European plant flowering phenology and flower-visiting insect activity phenology, we obtained plant and insect occurrence records from the GBIF (https://www.gbif.org/) for the period 2000–2024 within the study region (Lon: −15 to 36°, Lat: 32–72°). To improve the interpretability of dates and locations, we retained only human- and machine-observation records and excluded museum and herbarium specimen records. We further removed records with coordinate uncertainty >5 km to ensure adequate spatial precision. We selected plant records from angiosperms (classes Liliopsida and Magnoliopsida) and retained only observations explicitly annotated as ‘flowering’ to capture the availability of floral resources. For insects, we selected Lepidoptera records, a major flower-visiting group, and retained only adult-stage observations to represent the adult flight period. The Lepidoptera dataset included both butterflies and moths; however, we did not distinguish diurnal and nocturnal taxa because detailed time-of-day information was unavailable. We further excluded Lepidoptera taxa whose adult feeding habits were not associated with flower visitation, including non-feeding groups and other taxa not typically using floral resources as adult food sources, to better align occurrence records with floral resource use. Finally, to avoid structural bias in estimating within-year phenological metrics, we excluded non-seasonal taxa, including plants flowering year-round or across calendar years and Lepidoptera species flying year-round or showing clear long-distance migratory behaviour.

### (b) Flowering-plant and Lepidoptera phenological metric extraction

To quantify grid-scale phenological metrics of plant flowering and Lepidoptera flight activity, we discretized the study region into 1° × 1° latitude–longitude grid cells and extracted quantile-based phenological metrics. For phenological metrics estimation, we compiled the set of day-of-year (DOY) observations for each species *s* in grid cell *j* and year *t*, denoted {DOY}*_sjt_* and used it to estimate quantiles of the occurrence-time distribution. To ensure robust quantile estimation, we computed phenological metrics only for units meeting minimum sample-size requirements: each (*s*, *j*, *t*) combination had to include at least five observation records and cover at least three unique observation dates. We then estimated the 10th, 50th and 90th percentiles of the occurrence-time distribution using empirical quantiles, denoted *Q*_10_, *Q*_50_ and *Q*_90_, respectively. We further used *Q*_10_ as a proxy for onset and *Q*_90_ as a proxy for offset and defined the inter-quantile width *W* = *Q*_90_ − *Q*_10_ to represent the activity-window breadth. The resulting phenological metrics table, organized by (*s*, *j*, *t*) and including record counts, the number of unique dates and *Q*_10_, *Q*_50_, *Q*_90_ and *W*, served as an input for subsequent grid-scale trend modelling and spatial-gradient analyses.

Based on the above grid-scale phenological metrics, we further estimated phenological synchrony between flowering plants and Lepidoptera using an overlap-days metric. For each grid × year cell, we calculated the median *Q*_10_ and *Q*_90_ values separately for flowering plants and Lepidoptera to define their respective phenological windows, and we took the length of the intersection between these two windows as the overlap-days metric *D_jt_*.

### (c) Spatio-temporal trend modelling of flowering-plant and Lepidoptera phenology

To quantify spatio-temporal trends in plant flowering and Lepidoptera activity phenology during 2000–2024, we used linear mixed-effects models (LMMs). Based on the phenological metrics extraction described above, we compiled an annual phenological metrics dataset indexed by species × grid cell × year and retained only grid cells with at least 5 years of observation coverage within the study period to ensure robust trend estimation. We then fitted a unified LMM framework for each phenological pattern and its corresponding phenological metrics (*Q*_10_, *Q*_50_, *Q*_90_ and *W*), allowing estimation of the continental-scale (Europe-wide) mean rate of change and grid-scale (1° × 1°) rates of change. Specifically, let *M_sjt_* denote a given phenological metric for species *s* in grid cell *j* and year *t*. The model was specified as follows:

$$\;M_{sjt}=\beta_{0}\;+\beta_{1}\tilde{t}+b_{0j}+b_{1j}\tilde{t}+u_{0s}+\varepsilon_{sjt},$$

where $\tilde{t}=t-\bar{t}$ denotes the centred year, calculated by subtracting the mean year $\bar{t}$ from each year *t*; *β*_0_ and *β*_1_ denote the continental-scale intercept and mean rate of change (d yr⁻¹), respectively; *b_0j_* and *b_1j_* denote the random intercept and random slope for grid cell *j*, capturing spatial variation in baseline phenology and temporal trends; *u_0s_* denotes a species-level random intercept that accounts for systematic interspecific differences in baseline timing and *ε_sjt_* denotes the residual error term. Thus, *β*_1_ denotes the continental mean phenological change rate, while the grid-specific change rate for grid cell *j* is given by

$$\;R_{j}^{grid}=\beta_{1}\;+b_{1j}.$$

To quantify spatial trends in phenological change, we further applied ordinary least squares multiple regression. For each phenological metric (*Q*_10_, *Q*_50_, *Q*_90_ and *W*), we fitted the following model separately for flowering plants and Lepidoptera:

$$\;R_{j}^{grid}=\gamma_{0}\;+\gamma_{1}Lat_{j}+\gamma_{2}Lon_{j}+\eta_{j},$$

where Lat*_j_* and Lon*_j_* are the latitude and longitude of grid cell *j*, respectively, and *γ*_1_ and *γ*_2_ quantify the latitudinal and longitudinal gradients in phenological change. To account for multiple testing in the phenological trend and spatial-gradient analyses, we adjusted raw *P*-values using the Benjamini–Hochberg false discovery rate procedure; adjusted values are reported as *P*_BH_.

In addition, to assess the spatio-temporal variation in phenological synchrony between flowering plants and Lepidoptera, we analysed trends in the overlap-days metric *D_jt_*. Because this metric had already been aggregated to the grid × year level and no longer contained a species-level structure, we used regression models to characterize its overall temporal trend and spatial gradients. Let *D_jt_* denote the number of overlap days between flowering-plant and Lepidoptera phenology in grid cell *j* and year *t*. We specified the model as follows:

$$\;D_{jt}=\alpha_{0}\;+\alpha_{1}\tilde{t}+\alpha_{2}Lat_{j}+\alpha_{3}Lon_{j}+\alpha_{4}\left(\tilde{t}\times Lat_{j}\right)+\alpha_{5}\left(\tilde{t}\times Lon_{j}\right)+\varepsilon_{jt},$$

where $\tilde{t}=t-\bar{t}$ is the centred year; Lat*_j_* and Lon*_j_* are the latitude and longitude of grid cell *j*, respectively; *α_1_* is the overall temporal trend in phenological overlap; *α_2_* and *α_3_* are the main effects of latitude and longitude on phenological overlap, respectively; *α_4_* and *α_5_* are changes in the temporal trend along the latitudinal and longitudinal gradients, respectively and *ε_jt_* is the residual error term.

### (d) Analysis of the impact of environmental factors on flowering-plant and Lepidoptera phenology

To evaluate the effects of environmental factors on flowering-plant and Lepidoptera phenology, we derived long-term environmental data for Europe from 2000 to 2024 based on the E-OBS 32.0e dataset. E-OBS is a daily gridded observational data set for terrestrial Europe, constructed from the station network of the European Climate Assessment & Dataset project [38,39] and it is widely used as a key resource for climate monitoring across Europe [40]. In this study, we extracted mean air temperature (TEM), precipitation (PRE), mean wind speed (WIND), mean relative humidity (HU) and solar radiation (RAD) and aggregated the original 0.1° × 0.1° data to 1° × 1° monthly environmental variables. TEM, WIND, HU and RAD were aggregated as monthly means, whereas PRE was aggregated as monthly totals.

Because environmental conditions may affect biological phenology through cumulative pre-event effects, we used species × grid cell × year as the basic unit of analysis and extracted environmental conditions for the month of occurrence and the preceding six months for *Q*_10_, *Q*_50_ and *Q*_90_ [41]. To reduce bias arising from seasonal background differences in event-aligned environmental variables, we first calculated calendar-month anomalies for each environmental variable within each grid cell and then further removed their interannual linear trends. We then constructed candidate pre-season windows *w_0_* – *w_6_*. For mean-type variables (TEM, RAD, HU and WIND), each window variable was defined as the mean from the month of occurrence back to *k* months before the event; for the cumulative variable PRE, each window variable was defined as the cumulative total from the month of occurrence back to *k* months before the event.

To identify the optimal pre-season window for each environmental factor and phenological quantile, we fitted single-factor LMMs separately for *Q*_10_, *Q*_50_ and *Q*_90_ within each grid cell, using detrended phenological metrics as the response variable, the candidate-window environmental variable as a fixed effect and species as a random intercept:

$$\;Y_{sjt}^{*}=\theta_{0}\;+\theta_{1}X_{sjt}^{\left(k\right)}+u_{0s}+\varepsilon_{sjt},$$

where $Y_{sjt}^{*}$ denotes the detrended phenological metric of species *s* in grid cell *j* and year *t*, $X_{sjt}^{\left(k\right)}$ denotes the value of a given environmental factor under candidate window *w_k_*, *u_0s_* denotes the species-level random intercept and *ε_sjt_* denotes the residual error. The optimal pre-season window was selected as the one yielding the lowest Akaike information criterion.

Next, we used ridge regression (Ridge-R) as the main analytical framework to quantify the effects of environmental factors (TEM, PRE, WIND, HU and RAD) on the phenology of flowering plants and Lepidoptera, with phenological metrics as the response variables and the values of each environmental factor over its optimal pre-season window as predictors. Ridge-R [42] was applied to reduce the impact of multi-collinearity among predictors. In the ridge models, species terms were included together with the selected environmental predictors, and ridge regression coefficients (RCs) for the environmental variables were estimated using the R package ‘glmnet’ [43]. These coefficients represent the change in phenological timing associated with a 1 s.d. increase in the corresponding environmental factor. To corroborate the main patterns identified by Ridge-R, we further conducted partial correlation analysis, in which the association between a focal environmental factor and phenology was estimated while controlling for species effects and the other environmental predictors [44].

To quantify spatial gradients in environmental sensitivity, we treated the grid-specific ridge RCs for each environmental factor and phenological quantile (*Q*_10_, *Q*_50_ and *Q*_90_) as response variables and fitted separate ordinary least squares models against latitude and longitude:

$${R\;C}_{j}^{\left(e,q\right)}=\delta_{0}+\delta_{1}{\text{Lat}}_{j}+\varepsilon_{j}$$

and

$${R\;C}_{j}^{\left(e,q\right)}=\delta_{0}+\delta_{2}{\text{Lon}}_{j}+\varepsilon_{j},$$

where ${\text{RC}}_{j}^{\left(e,q\right)}$is the ridge RC of environmental factor *e* for phenological quantile *q* in grid cell *j*, and *δ_1_* and *δ_2_* are the latitudinal and longitudinal gradients in environmental sensitivity, respectively.

## 3. Results

### (a) Continental-scale overall trends in flowering-plant and Lepidoptera phenology across Europe

Using GBIF occurrence records from Europe for 2000–2024, we constructed a continent-wide phenological dataset for angiosperm flowering and adult Lepidoptera activity. After quality control and pre-processing, the final dataset included records for 707 angiosperm species and 620 Lepidoptera species. For species× grid cell × year units meeting the minimum sample-size requirements, we further extracted quantile-based phenological metrics, including *Q*_10_, *Q*_50_, *Q*_90_ and activity-window width (*W* = *Q*_90_ − *Q*_10_) (supplementary material, tables S1 and S2).

At the continental scale, our LMM showed that plant flowering phenology exhibited a pronounced early-season-driven pattern. Flowering onset (*Q*_10_) advanced significantly at a rate of −0.597 d yr⁻¹ (95% CI: [−0.818, −0.376] d yr⁻¹; *P* < 0.001), and the median flowering date (*Q*_50_) also advanced significantly at −0.507 d yr⁻¹ (95% CI: [−0.759, −0.254] d yr⁻¹; *P* < 0.001). By contrast, flowering termination (*Q*_90_) showed no significant change, with an estimated rate of −0.076 d yr⁻¹ (95% CI: [−0.284, −0.132] d yr⁻¹; *P* = 0.478) (figure 2a). Consistent with the stronger advance at the beginning of the season, the flowering activity window widened significantly (*Q*_90_ − *Q*_10_: +0.361 d yr⁻¹; 95% CI: [0.189,0.533] d yr⁻¹; *P* < 0.001), indicating that the flowering season at the European scale not only shifted earlier overall, but also became temporally extended in structure (figure 2b; supplementary material, table S3).

**Figure 2.**
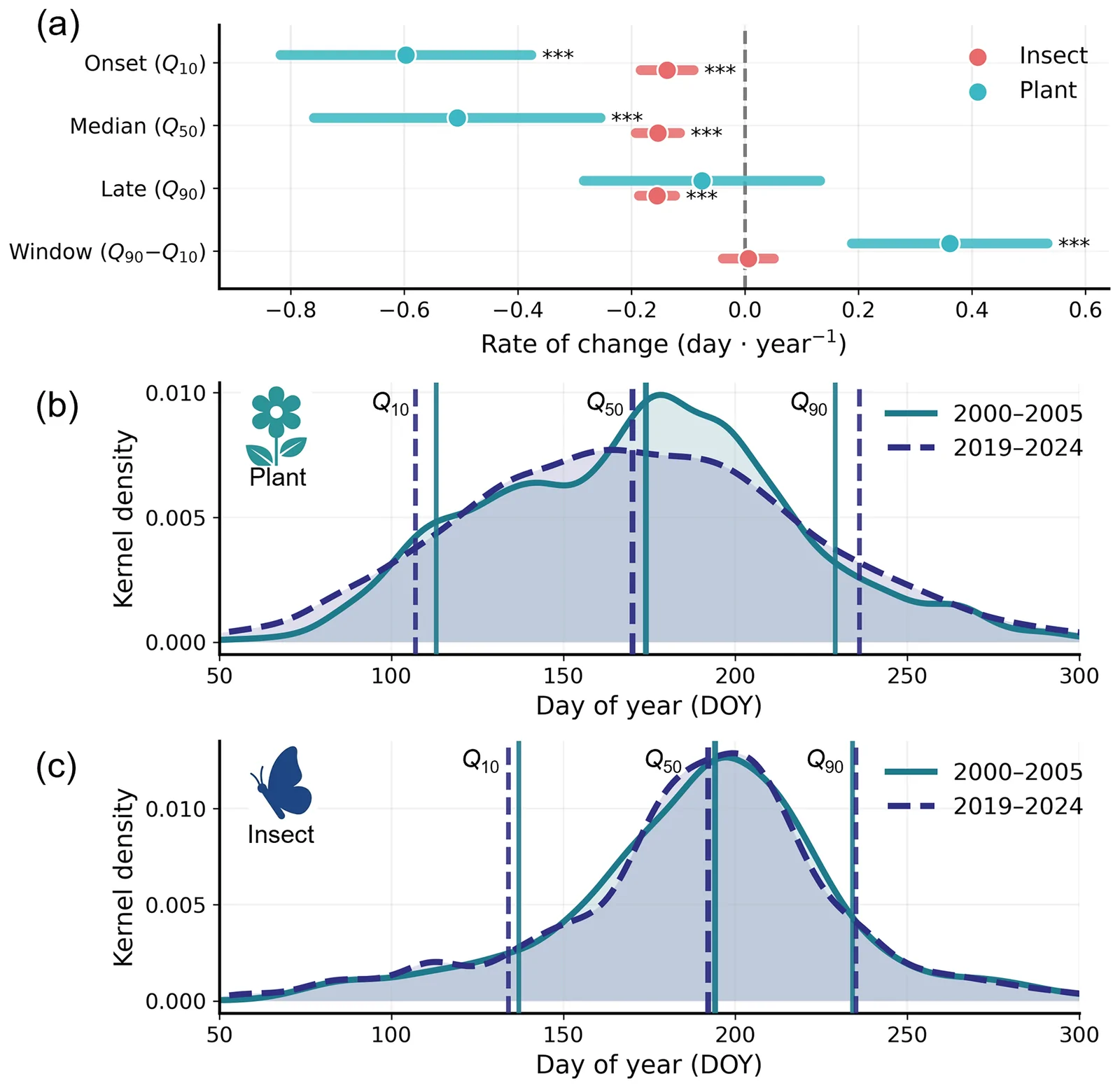
Flowering plants show stronger early-season advancement and significant phenological-window expansion, whereas Lepidoptera advance more uniformly across Europe during 2000–2024. (a) Phenological change rates of flowering plants and Lepidoptera during 2000–2024. Points indicate estimated change-rate coefficients and horizontal lines indicate 95% confidence intervals. (b) Distribution of plant flowering-day observation records across the calendar year during 2000–2005 and 2019–2024. (c) Distribution of Lepidoptera flight-period observation records across the calendar year during 2000–2005 and 2019–2024. Panels (b) and (c) are provided as illustrative examples of two contrasting periods. *Q*_10_: 10th percentile, *Q*_50_: 50th percentile, *Q*_90_: 90th percentile and window (*Q*_90_ − *Q*_10_) denotes phenological activity-window width. ***: *P* < 0.001.

Lepidoptera phenology, by contrast, showed a broadly coherent advance across all three quantiles. Flight-season onset (*Q*_10_) advanced significantly at −0.137 d yr⁻¹ (95% CI: [−0.184, −0.091] d yr⁻¹; *P* < 0.001), the median flight date (*Q*_50_) advanced at −0.153 d yr⁻¹ (95% CI: [−0.192, −0.114] d yr⁻¹; *P* < 0.001) and the end of the flight season (*Q*_90_) also advanced significantly, at −0.155 d yr⁻¹ (95% CI: [−0.187, −0.123] d yr⁻¹; *P* < 0.001) (figure 2a; supplementary material, table S4). However, the width of the activity window (*Q*_90_ − *Q*_10_) did not change significantly (+0.006 d yr⁻¹; 95% CI: [−0.039, 0.051] d yr⁻¹; *P* = 0.793), suggesting that Lepidoptera phenological responses were more consistent with a synchronous advance of the entire season rather than a marked restructuring of seasonal timing (figure 2c).

Further analyses showed that the number of phenological overlap days between flowering plants and Lepidoptera declined significantly over time (*α* = −0.340 d yr⁻¹, CI: [−0.636, −0.044] d yr⁻¹, *P* = 0.024) (supplementary material, table S5), indicating an overall weakening of phenological synchrony between the two groups during 2000–2024 and increasing mismatch at the continental scale.

### (b) Spatial trends in flowering-plant and Lepidoptera phenology across Europe

According to the regional-scale analyses (supplementary material, table S6), flowering-plant phenological trends showed limited variation along latitude and longitude, with spatial gradients detected for only a small subset of phenological metrics (figure 3a,b; supplementary material, table S7). Specifically, the latitudinal gradient of *Q*_10_ was +0.036 d yr⁻¹·°⁻¹ (95% CI: [0.001, 0.072]; *P* = 0.048), indicating that the advancing trend in flowering onset became slightly weaker with increasing latitude. By contrast, its longitudinal gradient was not significant (−0.022 d yr⁻¹·°⁻¹, 95% CI: [−0.050, 0.005]; *P* = 0.111). No significant latitudinal or longitudinal gradient was detected for *Q*_50_, with a latitudinal gradient of +0.021 d yr⁻¹·°⁻¹ (95% CI: [−0.018, 0.060]; *P* = 0.292) and a longitudinal gradient of −0.028 d yr⁻¹·°⁻¹ (95% CI: [−0.057, 0.001]; *P* = 0.063). Likewise, *Q*_90_ showed no significant trend along either latitude or longitude, with a latitudinal gradient of −0.001 d yr⁻¹·°⁻¹ (95% CI: [−0.022, 0.021]; *P* = 0.956) and a longitudinal gradient of −0.016 d yr⁻¹·°⁻¹ (95% CI: [−0.034, 0.002]; *P* = 0.08). The latitudinal gradient in window width was −0.012 d yr⁻¹·°⁻¹ (95% CI: [−0.025, 0.002]; *P* = 0.088), indicating only a weak tendency, whereas the longitudinal gradient was not significant (−0.006 d yr⁻¹·°⁻¹, 95% CI: [−0.022, 0.009]; *P* = 0.411). Overall, spatial gradients in flowering-plant phenological change were weak across Europe.

**Figure 3.**
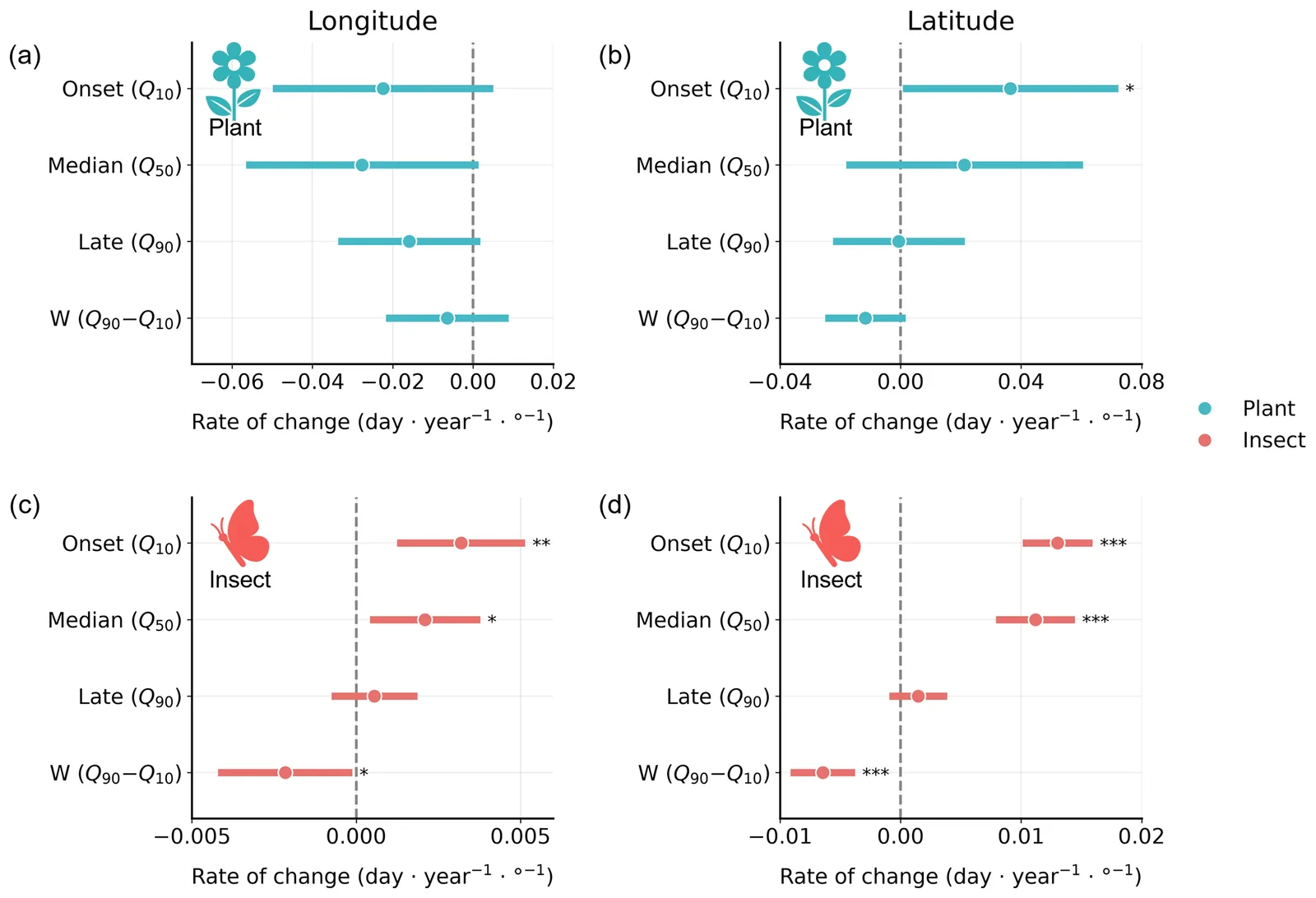
Lepidoptera phenological change exhibits clear latitudinal gradients, whereas flowering-plant phenological change remains spatially weak across Europe. (a) Longitudinal gradients for flowering plants. (b) Latitudinal gradients for flowering plants. (c) Longitudinal gradients for Lepidoptera. (d) Latitudinal gradients for Lepidoptera. Points indicate estimated gradient coefficients, representing changes in phenological change rates with longitude or latitude. Negative coefficients indicate a shift towards earlier phenology, whereas positive coefficients indicate a shift towards later phenology; horizontal lines indicate 95% confidence intervals. *Q*_10_: 10th percentile, *Q*_50_: 50th percentile, *Q*_90_: 90th percentile and W (*Q*_90_ − *Q*_10_) denotes phenological activity-window width. ***: *P* < 0.001; **: *P* < 0.01 and *: *P* < 0.05.

Lepidoptera, by contrast, exhibited clearer spatial trends, primarily in the latitudinal direction (figure 3c,d; supplementary material, table S7). Their latitudinal gradient of *Q*_10_ was +0.013 d yr⁻¹·°⁻¹ (95% CI: [0.010, 0.016]; *P* < 0.001) and that of *Q*_50_ was +0.011 d yr⁻¹·°⁻¹ (95% CI: [0.008, 0.014]; *P* < 0.001), indicating that the advancing trends in flight onset and median timing became progressively weaker towards higher latitudes. The longitudinal gradients of Lepidoptera responses were also significant, with *Q*_10_ at +0.003 d yr⁻¹·°⁻¹ (95% CI: [0.001, 0.005]; *P* = 0.001) and *Q*_50_ at +0.002 d yr⁻¹·°⁻¹ (95% CI: [0.0004, 0.004]; *P* = 0.015). By contrast, *Q*_90_ showed no significant latitudinal or longitudinal gradient, with a latitudinal gradient of +0.001 d yr⁻¹·°⁻¹ (95% CI: [−0.001, 0.004]; *P* = 0.24) and a longitudinal gradient of +0.001 d yr⁻¹·°⁻¹ (95% CI: [−0.001, 0.002]; *P* = 0.406). Window width showed a significant latitudinal contraction pattern, with a gradient of −0.006 d yr⁻¹·°⁻¹ (95% CI: [−0.009, −0.004]; *P* < 0.001), indicating that Lepidoptera activity windows narrowed more strongly at higher latitudes. Its longitudinal gradient was −0.002 d yr⁻¹·°⁻¹ (95% CI: [−0.004, −0.0001]; *P* = 0.039), suggesting a slight additional tendency towards window narrowing from west to east.

Further analysis of phenological overlap days between flowering plants and Lepidoptera showed no significant main effects of latitude or longitude, whereas the year × latitude interaction was positive and marginally significant (*α* = 0.055, *P* = 0.092) (supplementary material, table S5). This suggests that the decline in phenological overlap over time weakened towards higher latitudes but was faster at lower latitudes, where mismatch increased more strongly. However, these spatial gradients should be interpreted cautiously because the estimated effect sizes were generally small, and statistical significance may be partly influenced by sample size.

### (c) Environmental drivers and divergent responses of flowering-plant and Lepidoptera phenology

Differences in the rates of phenological change between flowering plants and Lepidoptera may arise from differences in the direction, magnitude and stage dependence of their responses to the same environmental drivers. Ridge-R showed that the mean *R*^2^ values for *Q*_10_, *Q*_50_ and *Q*_90_ in flowering plants were 0.22, 0.18 and 0.19, respectively, whereas the corresponding values for Lepidoptera were 0.30, 0.30 and 0.24. This indicates that, within the current modelling framework, the selected environmental factors explained a greater proportion of phenological variation in Lepidoptera than in flowering plants.

Further analysis showed that flowering-plant phenology exhibited clear stage dependence in its responses to environmental factors (figure 4a–c). Temperature was the dominant driver of flowering advancement, but its effect weakened progressively across phenological stages. The median standardized ridge coefficients of TEM were −2.83, −2.62 and −1.95 for *Q*_10_, *Q*_50_ and *Q*_90_, respectively. The effect of WIND changed direction across phenological stages: it showed a relatively pronounced advancing effect at *Q*_10_ (−0.52), was close to neutral at *Q*_50_ (−0.05) and shifted to a delaying effect at *Q*_90_ (+0.45). RAD showed an overall weak advancing effect, with median ridge coefficients of −0.13, −0.24 and −0.24, respectively. By contrast, moisture-related factors generally showed delaying effects, with median ridge coefficients for HU of +0.32, +0.35 and +0.15 for *Q*_10_, *Q*_50_ and *Q*_90_, respectively, and those for PRE of +0.01, +0.15 and +0.21. Partial-correlation analysis was broadly consistent with the Ridge-R results (supplementary material, figure S2), with temperature showing the largest absolute partial-correlation coefficients, further indicating that flowering phenology was primarily driven by temperature. The independent effects of other climatic factors were relatively weak and showed marked fluctuations across phenological stages.

**Figure 4.**
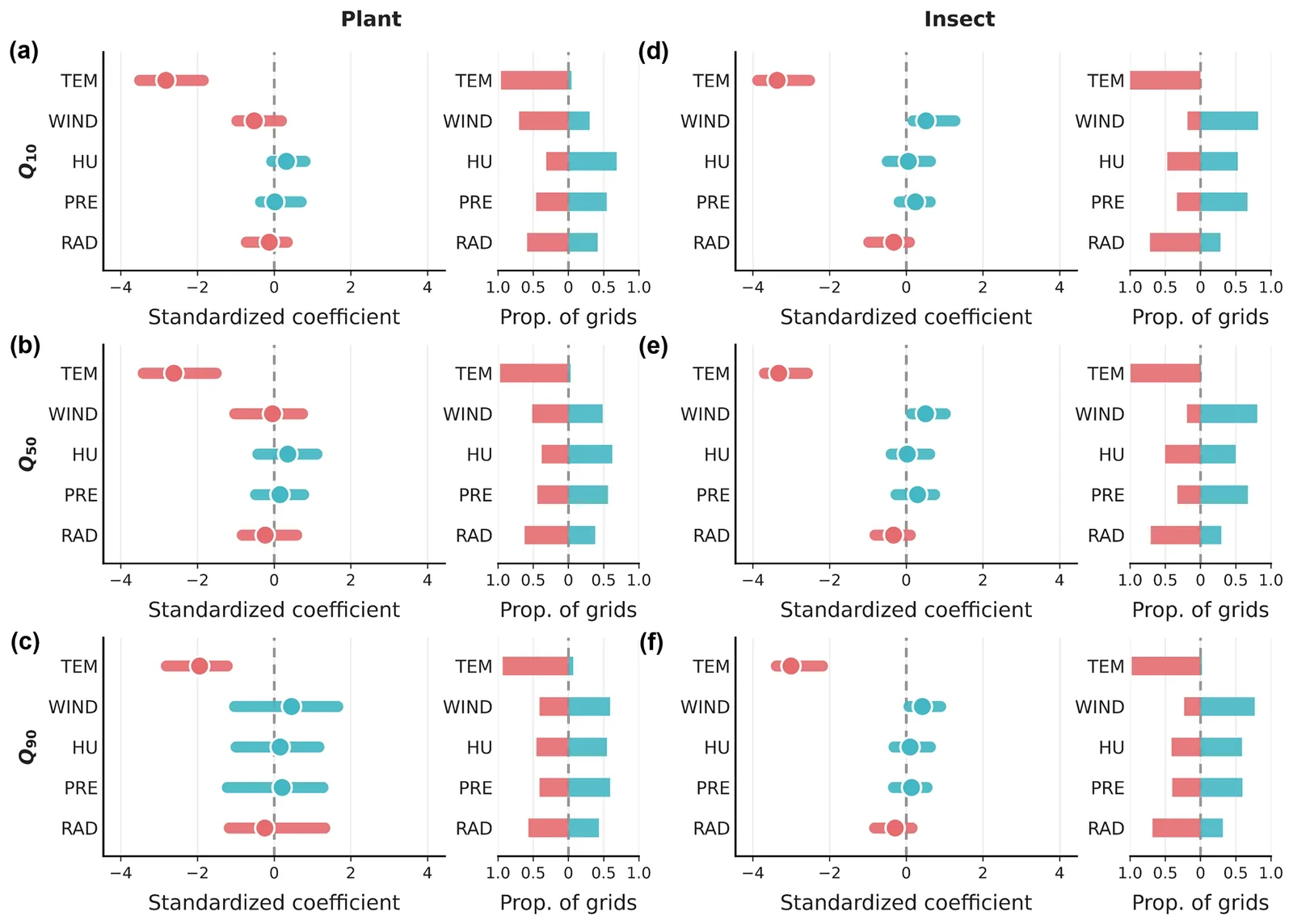
Flowering plants show more stage-dependent environmental responses, whereas Lepidoptera respond more consistently across phenological stages. (a–c) *Q*_10_, *Q*_50_ and *Q*_90_ for flowering plants. (d–f) *Q*_10_, *Q*_50_ and *Q*_90_ for Lepidoptera. Dots and horizontal bars indicate median standardized ridge coefficients and interquartile ranges (25th–75th percentiles), respectively; right-hand bars show the proportion of grids with negative and positive effects. Negative coefficients indicate phenological advancement (red), whereas positive coefficients indicate delay (blue).

In addition, Lepidoptera phenology showed a comparatively stable response to environmental factors, with relatively small variation across stages (figure 4d–f). TEM was likewise the dominant driver of phenological advancement, with median standardized ridge coefficients of −3.37, −3.33 and −3.01 for *Q*_10_, *Q*_50_ and *Q*_90_, respectively. WIND showed delaying effects across all three phenological stages, with median ridge coefficients of +0.51, +0.50 and +0.42. RAD showed a weak but stable advancing effect overall, with median ridge coefficients of −0.33, −0.33 and −0.29. Similarly, moisture-related factors generally showed delaying effects, with median ridge coefficients for HU of +0.05, +0.02 and +0.10 for *Q*_10_, *Q*_50_, and *Q*_90_, respectively, and those for PRE of +0.24, +0.29, and +0.13. Partial-correlation analysis was also broadly consistent with the Ridge-R results (supplementary material, figure S2), with temperature showing the largest absolute partial-correlation coefficients, further indicating that Lepidoptera phenology was primarily driven by temperature. At the same time, the directions of the effects of wind speed and solar radiation were also largely consistent between the two methods, whereas the independent effects of humidity and precipitation were relatively weak.

To test whether environmental sensitivity of phenology varied systematically across space, we further examined latitudinal and longitudinal gradients in the estimated sensitivity coefficients (supplementary material, table S8). Overall, spatial gradients in flowering-plant phenological sensitivity were weak (figure 5). For most environmental factors, slopes along both latitude and longitude were not significant, with only a few exceptions for PRE, TEM and RAD. Among these, PRE sensitivity showed the clearest and most consistent pattern across all three phenological quantiles. At *Q*_10_, the latitudinal and longitudinal slopes were *δ*_lat_ = −0.104 (*P* = 0.017) and *δ*_lon_ = −0.103 (*P* < 0.001), respectively; at *Q*_50_, they were *δ*_lat_ = −0.156 (*P* < 0.001) and *δ*_lon_ = −0.071 (*P* = 0.021); and at *Q*_90_, they were *δ*_lat_ = −0.154 (*P* = 0.026) and *δ*_lon_ = −0.175 (*P* < 0.001). These results indicate that PRE sensitivity on flowering-plant phenology declined with increasing latitude and longitude, implying a stronger delaying effect of precipitation in Southern and Western Europe. By contrast, TEM sensitivity showed a significant positive longitudinal gradient only at *Q*_90_ (*δ*_lon_ = +0.093, *P* = 0.010), whereas RAD sensitivity showed a significant negative longitudinal gradient only at *Q*_10_ (*δ*_lon_ = −0.061, *P* = 0.014).

**Figure 5.**
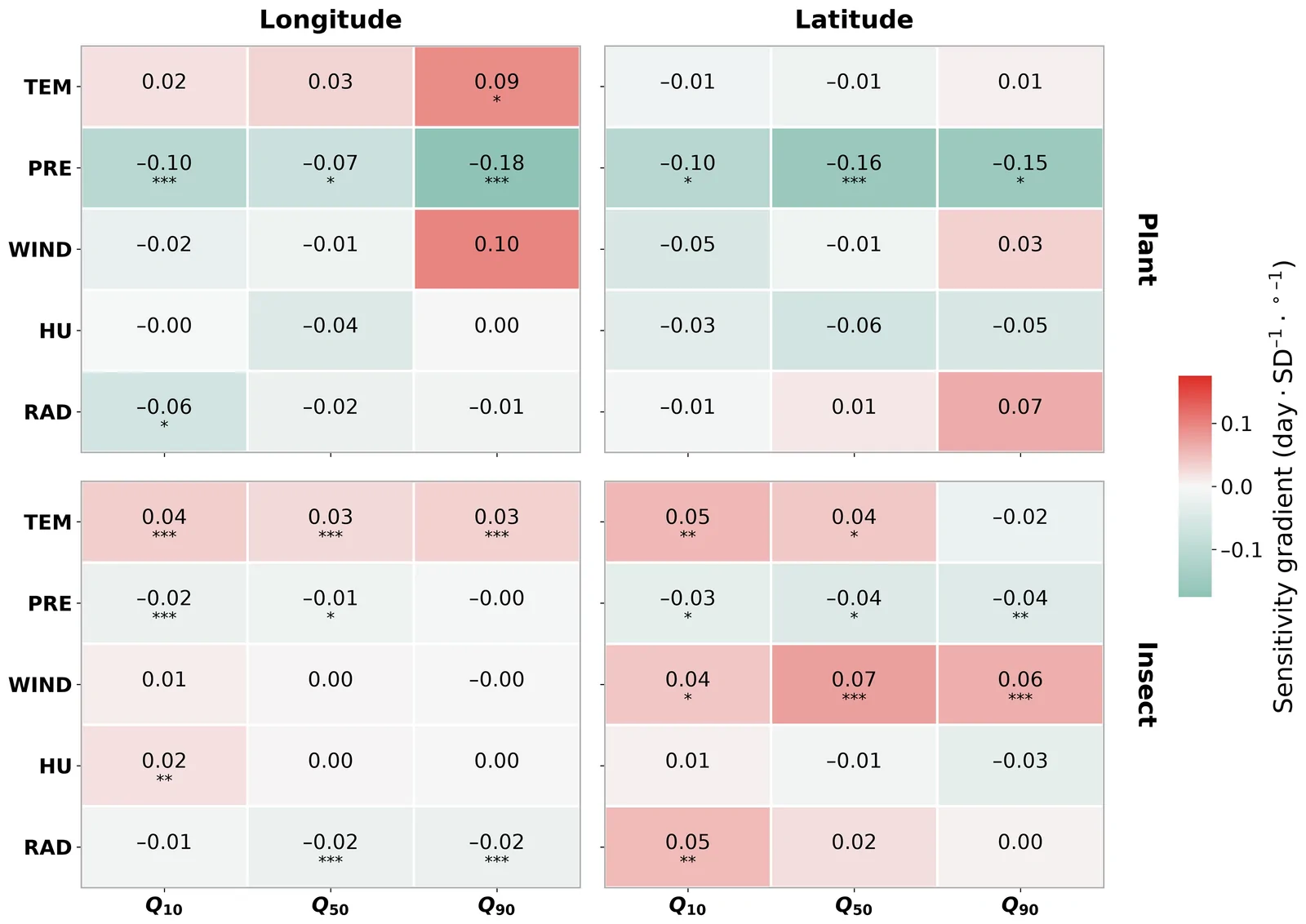
Environmental sensitivity is more spatially structured in Lepidoptera than in flowering plants across Europe. The heat maps show the spatial gradients in the sensitivity of flowering-plant and Lepidoptera phenology to the main environmental factors (TEM, PRE, WIND, HU and RAD) across different phenological stages (*Q*_10_, *Q*_50_ and *Q*_90_). Values within cells indicate the regression slope *δ*, representing how the phenological effect of each environmental factor changes with longitude or latitude. Negative slopes indicate that the effect of increasing the environmental factor shifts towards phenological advancement, whereas positive slopes indicate a shift towards phenological delay. *, ** and *** indicate significance at *P* < 0.05, *P* < 0.01 and *P* < 0.001, respectively.

By contrast to flowering plants, Lepidoptera phenological sensitivity exhibited a clearer spatial structure, with more consistent gradients across multiple environmental factors (figure 5). TEM sensitivity showed significant positive latitudinal gradients at both *Q*_10_ (*δ*_lat_ = +0.054, *P* = 0.004) and *Q*_50_ (*δ*_lat_ = +0.040, *P* = 0.031), indicating that the advancing effect of temperature on Lepidoptera phenology weakened towards higher latitudes. Along the longitudinal axis, TEM sensitivity was also significantly positive at *Q*_10_ (*δ*_lon_ = +0.036, *P* < 0.001), *Q*_50_ (*δ*_lon_ = +0.025, *P* < 0.001) and *Q*_90_ (*δ*_lon_ = +0.032, *P* < 0.001), suggesting that the temperature-driven advance similarly weakened towards the east. PRE sensitivity showed significant negative latitudinal gradients at *Q*_10_ (*δ*_lat_ = −0.027, *P* = 0.045) and *Q*_50_ (*δ*_lat_ = −0.044, *P* = 0.017), and a significant negative longitudinal gradient at *Q*_10_ (*δ*_lon_ = −0.020, *P* < 0.001), indicating that the delaying effect of precipitation became weaker with increasing latitude and longitude. WIND sensitivity showed significant positive latitudinal gradients at *Q*_10_ (*δ*_lat_ = +0.042, *P* = 0.015), *Q*_50_ (*δ*_lat_ = +0.075, *P* < 0.001) and *Q*_90_ (*δ*_lat_ = +0.063, *P* < 0.001), implying that the delaying effect of wind strengthened towards higher latitudes. RAD sensitivity displayed a positive latitudinal gradient at *Q*_10_ (*δ*_lat_ = +0.053, *P* = 0.003), but significant negative longitudinal gradients at *Q*_50_ (*δ*_lon_ = −0.022, *P* < 0.001) and *Q*_90_ (*δ*_lon_ = −0.023, *P* < 0.001). Humidity sensitivity was significant only at *Q*_10_, where it showed a positive longitudinal gradient (*δ*_lon_ = +0.020, *P* = 0.009), but was otherwise non-significant.

## 4. Discussion

The extent and direction of changes in phenological synchrony are the outcome of intricate interactions between the life history strategies of plants and insects [45], their physiological characteristics [11] and external environmental factors [46]. Using GBIF occurrence records from 2000 to 2024, we compared the flowering phenology of 707 angiosperm species with the adult activity phenology of 620 Lepidoptera species across Europe and found a progressive decline in the temporal overlap between the two groups. This increasing mismatch appears to be driven primarily by the more rapid advance of flowering-plant phenology, especially during the early and middle parts of the growing season, whereas Lepidoptera phenology advanced more evenly across the season. Taken together, these findings indicate that the temporal linkage between flowering plants and flower-visiting Lepidoptera has weakened across Europe during 2000–2024. This pattern is broadly consistent with previous studies [37,47,48] and suggests that weakening phenological synchrony may further affect pollination interactions and related ecosystem functions.

Flowering plants and Lepidoptera differed markedly in the structure of their phenological sensitivities to environmental drivers. Overall, the two groups showed broadly consistent response directions to temperature, solar radiation, humidity and precipitation but differed in the strength and stage dependence of these effects (figure 4). Temperature was the dominant driver of phenological advance in both flowering plants and Lepidoptera. This is probably because warming accelerates dormancy release, floral bud development and heat accumulation in plants [49,50], while also increasing metabolic rate and developmental speed in insects, thereby promoting emergence and adult activity and jointly advancing phenology [51]. Solar radiation also showed an overall advancing effect, which may be related to its role in enhancing photosynthesis, increasing available energy input and improving near-surface thermal conditions [52,53]. By contrast, precipitation and humidity generally exerted delaying effects. In plants, wetter conditions are often associated with increased cloud cover, reduced radiation and lower effective heat accumulation, thereby constraining the progression of flowering phenology [54]. In insects, higher humidity and precipitation may not only alter developmental rhythms, but also suppress flight, feeding and mate-searching activity, causing insect phenology to appear later overall [55]. Unlike the factors above, wind speed showed contrasting effects on flowering-plant and Lepidoptera phenology. In flowering plants, wind tended to promote phenology during the early and middle stages of flowering but shifted to a delaying effect in the late stage, indicating clear stage dependence. One possible explanation is that stronger winds earlier in the season may be associated with warm-air advection and enhanced heat accumulation, thereby promoting floral development and the onset of flowering. By contrast, stronger winds later in the season may more often coincide with low-temperature, low-radiation weather conditions, which could delay late-season phenology [56]. In Lepidoptera, however, wind speed showed an overall delaying effect, more probably reflecting the constraining effects of wind on flight stability, foraging efficiency and the probability of adult activity [57].

Further analyses revealed marked differences in the geographical structure of phenological change between flowering plants and Lepidoptera. Across Europe, changes in flowering phenology were spatially relatively uniform, with only a slight weakening in the advance of flowering onset towards higher latitudes, whereas no significant spatial gradients were detected for the other phenological metrics (figure 3). By contrast, Lepidoptera phenology exhibited a clearer latitudinal structure: the advancing trends in both flight-season onset and mid-season timing weakened significantly with increasing latitude, and the activity window also became progressively narrower (figure 3). This pattern closely matched the spatial structure of Lepidoptera temperature sensitivity, which declined significantly towards higher latitudes and longitudes (figure 5), and may reflect stronger diapause dependence and greater thermal requirements at high latitudes, both of which could constrain the magnitude of phenological responses to warming [51,58]. Unlike Lepidoptera, flowering-plant phenology showed no clear geographical structuring in its sensitivity to environmental drivers. The only consistent pattern was that the delaying effect of precipitation was stronger in southern and western Europe (figure 5), which is consistent with stronger water limitation on late-season flowering under the dry summer conditions characteristic of the Mediterranean climate region [54].

In recent years, open-access datasets containing long-term records of plant and insect phenology have become increasingly available, and the large volume of species-occurrence data aggregated by platforms, such as GBIF, has provided an important basis for phenological research at continental scales [59,60]. However, results derived from such datasets should be interpreted with caution, as they inevitably contain multiple sources of bias [61–64]. First, GBIF records are not collected under standardized sampling schemes, and observation intensity varies substantially among countries, years and species. As a result, continent-scale estimates of phenological trends may be geographically biased towards regions with denser records. Second, the activity of citizen-science observers is itself seasonally concentrated, and if the seasonal distribution of recording effort changes systematically over time, this may introduce trend signals into phenological metrics that do not reflect underlying biological change. Nevertheless, by using quantile-based phenological metrics, applying minimum sample-size thresholds and adopting a mixed-effects modelling framework to account for systematic differences among species and across space, our study probably reduced the influence of these biases on trend estimation to some extent. In addition, phenological overlap inferred from occurrence records may not directly translate into realized interactions or fitness consequences, because plant–insect relationships can differ in host specialization and interaction strength among species.

The progressive decline in phenological overlap between flowering plants and flower-visiting Lepidoptera reported here, if sustained, may have far-reaching consequences for pollination networks, food webs and ecosystem services [20,25,65]. Phenological asynchrony between flowering plants and their flower-visiting insects has already been shown to influence population dynamics [66,67], adaptive capacity [68] and species interactions [24]. A clearer understanding of the physiological and ecological mechanisms underlying the contrasting phenological responses of plants and insects would therefore substantially improve our ability to predict and potentially mitigate the consequences of phenological mismatch [69]. Future research should place greater emphasis on the complex interactions within interspecific phenological networks and on the combined roles of biotic and abiotic factors in shaping phenological change [18,70,71]. In particular, more attention should be given to the phenological changes of specific plant–insect pairs, as well as to the effects of environmental change on their physiological and ecological traits. Such efforts will require more detailed physiological, ecological and life-history data across species and across broad spatial extents, including more explicit information on life stages, feeding relationships and key phenological events. Advancing this line of research will greatly improve our understanding of the patterns and consequences of phenological asynchrony in regions experiencing intensified climate change. More broadly, such large-scale integrative approaches will provide a stronger basis for understanding and managing phenological mismatch, thereby supporting the stability of ecosystems and biodiversity.

## Supplementary Material



## Data Availability

All data used in this analysis are publicly accessible. Original occurrence records were obtained from Global Biodiversity Information Facility (Lepidoptera: https://doi.org/10.15468/dl.432b9d, Liliopsida: https://doi.org/10.15468/dl.b3abj7 and Magnoliopsida: https://doi.org/10.15468/dl.9nwvfk). E-OBS data were obtained from the Copernicus Climate Change Service Climate Data Store [38,39]. The processed data, analysis outputs and associated code are available in the Zenodo repository [72]. Supplementary material is available online [73].
