# Supplementary material for: Phenological divergence between flowering plants and flower-visiting Lepidoptera under climate change across Europe
Source: Proc Biol Sci. 2026 Nov 4;293(2082):20261381. doi: 10.1098/rspb.2026.1381 (PMC13730798; doi:10.1098/rspb.2026.1381)
Supplement: Supplementary file 1 [file rspb.2026.1381.zip › rspb.2026.1381/rspb20261381_si_002.docx]

Supplementary Materials for

**Phenological divergence between flowering plants and flower-visiting Lepidoptera under climate change across Europe**

Yanru Huang, Chaoyang Wu^*^, Wenjiang Huang^*^, Yang Liu, Man Qi, Jie Bai, Yingying Dong, Samuel J L Gascoigne, Philippe Ciais, Josep Peñuelas, Roberto Salguero-Gómez^*^

**This PDF file includes:**

Tables S1 to S8 (Table S1, S2 and S6 are provided in additional form)

Figures. S1 to S2

**Table S3**. Continental-scale phenological change rates of flowering plants across Europe during 2000–2024

| Metric | Continental-scale slope (${d\cdot yr}^{-1}$) | CI_2.5%_ | CI_97.5%_ | *P* | *P*_BH_ |
| --- | --- | --- | --- | --- | --- |
| Q_10_ | -0.597 | -0.818 | -0.376 | 6.62E-07 | 2.65E-06 |
| Q_50_ | -0.507 | -0.759 | -0.254 | 1.56 E-04 | 0.000208 |
| Q_90_ | -0.076 | -0.284 | 0.132 | 0.478 | 0.478 |
| Width (Q_90_-Q_10_) | 0.361 | 0.189 | 0.533 | 9.22E-05 | 1.84 E-04 |

**P*_BH_ denotes the Benjamini–Hochberg adjusted *P*-value.

**Table S4**. Continental-scale phenological change rates of Lepidoptera across Europe during 2000–2024

| Metric | Continental-scale slope (${d\cdot yr}^{-1}$) | CI_2.5%_ | CI_97.5%_ | *P* | *P*_BH_ |
| --- | --- | --- | --- | --- | --- |
| Q_10_ | -0.137 | -0.184 | -0.091 | 6.20E-08 | 8.27E-08 |
| Q_50_ | -0.153 | -0.192 | -0.114 | 4.10E-12 | 8.20E-12 |
| Q_90_ | -0.155 | -0.187 | -0.123 | 2.31E-15 | 9.23E-15 |
| Width (Q_90_-Q_10_) | 0.006 | -0.039 | 0.051 | 0.793 | 0.793 |

**P*_BH_ denotes the Benjamini–Hochberg adjusted *P*-value.

**Table S5**. Spatiotemporal variation in phenological overlap days between flowering plants and Lepidoptera

| Parameter | Estimate | SE | *P* | 95% CI |
| --- | --- | --- | --- | --- |
| Year | -0.340 | 0.151 | 0.024 | [-0.636, -0.044] |
| latitude | 0.044 | 0.146 | 0.763 | [-0.242, 0.330] |
| longitude | -0.126 | 0.087 | 0.144 | [-0.296, 0.043] |
| Year × latitude | 0.055 | 0.033 | 0.092 | [-0.009, 0.119] |
| Year ×longitude | -0.003 | 0.017 | 0.874 | [-0.036, 0.031] |

**Table S7**. Latitudinal and longitudinal gradients in flowering-plant and Lepidoptera phenological trends across Europe

| Dataset | Metric | Slope_lat_ | CI_lat_95%_ | Slope_lon_ | CI_lon_95%_ | *P*_lat_ | *P*_lon_ | *P*_lat_BH_ | *P*_lon_BH_ |
| --- | --- | --- | --- | --- | --- | --- | --- | --- | --- |
| Plant | Q_10_ | 0.036 | [0.001, 0.072] | -0.022 | [-0.050, 0.005] | 0.048 | 0.111 | 0.176 | 0.148 |
|  | Q_50_ | 0.021 | [-0.018, 0.060] | -0.028 | [-0.057, 0.001] | 0.292 | 0.063 | 0.390 | 0.148 |
|  | Q_90_ | -0.001 | [-0.022, 0.021] | -0.016 | [-0.034, 0.002] | 0.956 | 0.080 | 0.956 | 0.148 |
|  | Width (Q_90_-Q_10_) | -0.012 | [-0.025, 0.002] | -0.006 | [-0.022, 0.009] | 0.088 | 0.411 | 0.176 | 0.411 |
| Insects | Q_10_ | 0.013 | [0.010, 0.016] | 0.003 | [0.001, 0.005] | 1.12E-16 | 0.001 | 4.50E-16 | 0.006 |
|  | Q_50_ | 0.011 | [0.008, 0.014] | 0.002 | [0.0004, 0.004] | 1.08E-10 | 0.015 | 2.16E-10 | 0.030 |
|  | Q_90_ | 0.001 | [-0.001, 0.004] | 0.001 | [-0.001, 0.002] | 0.240 | 0.406 | 0.240 | 0.406 |
|  | Width (Q_90_-Q_10_) | -0.006 | [-0.009, -0.004] | -0.002 | [-0.004, 0.0001] | 3.10E-06 | 0.039 | 4.14E-06 | 0.052 |

**P*_BH_ denotes the Benjamini–Hochberg adjusted *P*-value.

**Table S8**. Latitudinal and longitudinal gradients in the environmental sensitivity of flowering-plant and Lepidoptera phenology across Europe

| Dataset | Metric | Environmental factors | Slope_lat_ | SE_lat | *P* | Slope_lon_ | SE_lon | *P* |
| --- | --- | --- | --- | --- | --- | --- | --- | --- |
| Plant | Q_10_ | WIND | -0.052 | 0.057 | 0.362 | -0.024 | 0.040 | 0.545 |
|  |  | HU | -0.035 | 0.030 | 0.253 | -0.002 | 0.021 | 0.935 |
|  |  | RAD | -0.007 | 0.036 | 0.850 | -0.061 | 0.024 | 0.014 |
|  |  | PRE | -0.104 | 0.042 | 0.017 | -0.103 | 0.029 | 0.001 |
|  |  | TEM | -0.010 | 0.043 | 0.817 | 0.018 | 0.030 | 0.543 |
|  | Q_50_ | WIND | -0.014 | 0.040 | 0.738 | -0.014 | 0.031 | 0.650 |
|  |  | HU | -0.061 | 0.043 | 0.160 | -0.044 | 0.033 | 0.185 |
|  |  | RAD | 0.013 | 0.045 | 0.775 | -0.017 | 0.034 | 0.617 |
|  |  | PRE | -0.156 | 0.036 | <0.001 | -0.071 | 0.030 | 0.021 |
|  |  | TEM | -0.015 | 0.036 | 0.685 | 0.029 | 0.027 | 0.283 |
|  | Q_90_ | WIND | 0.033 | 0.078 | 0.675 | 0.098 | 0.053 | 0.071 |
|  |  | HU | -0.051 | 0.060 | 0.399 | <0.001 | 0.043 | 0.996 |
|  |  | RAD | 0.065 | 0.074 | 0.382 | -0.012 | 0.053 | 0.826 |
|  |  | PRE | -0.154 | 0.066 | 0.026 | -0.175 | 0.042 | <0.001 |
|  |  | TEM | 0.005 | 0.053 | 0.918 | 0.093 | 0.034 | 0.010 |
| Insect | Q_10_ | WIND | 0.042 | 0.017 | 0.015 | 0.009 | 0.007 | 0.199 |
|  |  | HU | 0.006 | 0.020 | 0.759 | 0.020 | 0.008 | 0.009 |
|  |  | RAD | 0.053 | 0.018 | 0.003 | -0.009 | 0.007 | 0.186 |
|  |  | PRE | -0.027 | 0.014 | 0.045 | -0.020 | 0.005 | <0.001 |
|  |  | TEM | 0.054 | 0.018 | 0.004 | 0.036 | 0.007 | <0.001 |
|  | Q_50_ | WIND | 0.074 | 0.019 | <0.001 | 0.002 | 0.008 | 0.783 |
|  |  | HU | -0.008 | 0.019 | 0.684 | 0.003 | 0.007 | 0.721 |
|  |  | RAD | 0.021 | 0.017 | 0.226 | -0.022 | 0.006 | 0.001 |
|  |  | PRE | -0.044 | 0.018 | 0.017 | -0.015 | 0.007 | 0.038 |
|  |  | TEM | 0.040 | 0.019 | 0.031 | 0.025 | 0.007 | <0.001 |
|  | Q_90_ | WIND | 0.063 | 0.017 | <0.001 | <0.001 | 0.007 | 0.956 |
|  |  | HU | -0.029 | 0.017 | 0.085 | 0.001 | 0.006 | 0.921 |
|  |  | RAD | 0.004 | 0.016 | 0.786 | -0.023 | 0.006 | 0.000 |
|  |  | PRE | -0.040 | 0.015 | 0.007 | -0.004 | 0.006 | 0.544 |
|  |  | TEM | -0.019 | 0.021 | 0.376 | 0.032 | 0.008 | 0.000 |

*Abbreviations: TEM, mean air temperature; PRE, precipitation; WIND, mean wind speed; HU, mean relative humidity; and RAD, solar radiation.

**Figure S1.** Workflow used to analyse potential phenological changes in flowering plants and Lepidoptera. (1) Data acquisition and preprocessing, including three types of data: environmental, Lepidoptera, and plant data. (2) Spatiotemporal trend analysis of phenological changes. A linear mixed-effects model (LMM) incorporating both continental-scale and grid-scale effects was constructed, and grid-level phenological change rates were further regressed against latitude and longitude to quantify geographic gradients. (3) Assessment of environmental drivers of phenology: partial correlation analysis and ridge regression were applied to quantify the influence of environmental variables.

**Figure S2.** Environmental effects on flowering-plant and Lepidoptera phenology across Europe based on partial correlation analysis. (a–c) $Q_{10}$, $Q_{50}$, and $Q_{90}$ for flowering plants. (d–f) $Q_{10}$, $Q_{50}$, and $Q_{90}$ for Lepidoptera. Dots and horizontal bars indicate median partial correlation coefficients and interquartile ranges (25^th^–75^th^ percentiles), respectively; right-hand bars show the proportion of grids with significantly negative and positive effects (*P* < 0.05). Negative coefficients indicate phenological advancement (red), whereas positive coefficients indicate delay (blue).
